# The Future of Hyperspectral Imaging

**DOI:** 10.3390/jimaging5110084

**Published:** 2019-10-25

**Authors:** Stefano Selci

**Affiliations:** Institute for Photonics and Nanotechnologies, ARTOV C.N.R., Via del Fosso del Cavaliere 100, 00133 Roma, Italy; Stefano.Selci@cnr.it

**Keywords:** hyperspectral imaging, medical imaging by HSI, HSI for biology, remote sensing, hyperspectral microscopy, fluorescence hyperspectral imaging, Raman hyperspectral imaging, infrared hyperspectral imaging, statistical methods for HSI, hyperspectral data mining and compression, statistical methods for HSI, hyperspectral data mining and compression

## Abstract

The Special Issue on hyperspectral imaging (HSI), entitled “The Future of Hyperspectral Imaging”, has published 12 papers. Nine papers are related to specific current research and three more are review contributions: In both cases, the request is to propose those methods or instruments so as to show the future trends of HSI. Some contributions also update specific methodological or mathematical tools. In particular, the review papers address deep learning methods for HSI analysis, while HSI data compression is reviewed by using liquid crystals spectral multiplexing as well as DMD-based Raman spectroscopy. Specific topics explored by using data obtained by HSI include alert on the sprouting of potato tubers, the investigation on the stability of painting samples, the prediction of healing diabetic foot ulcers, and age determination of blood-stained fingerprints. Papers showing advances on more general topics include video approach for HSI dynamic scenes, localization of plant diseases, new methods for the lossless compression of HSI data, the fusing of multiple multiband images, and mixed modes of laser HSI imaging for sorting and quality controls.

## 1. Introduction to This Special Issue

Hyperspectral imaging (HSI) manages to gather images where any single pixel is associated a full spectrum in a given range. Unlike multispectral techniques that are already capable of acquiring similar data within a few spectral bands, a continuous spectrum is available through HSI. As research on specific materials and their properties is commonly identified by spectral signatures, HSI exploits the same objective down to the pixel level with the further advantage of possible classification and segmentation of the overall spectral and imaging information, like for land analysis by satellite observation showing distinct regions with water, mineral resources, or plant extensions. No prior knowledge is usually needed except for what can be obtained exclusively from the dataset.

The remarkable mix of information is often represented by “hypercubes”, a multidimensional representation of the obtained data along multiple axes providing a picture of the spatial distribution of the observed information: Spectroscopic (one axis, with signal coming from reflectance, fluorescence, Raman, or any other spectroscopic probe), structural (three axes), and also time (a possible further axis).

A single image pixel can range down to microscopic detail and, for HSI-based microscopes, up to meters for satellite data. It should be noted that the spectral component does not have to be limited to the visible range, for instance, the infrared spectral region can be a common choice.

The rapid increase of possible HSI applications and the availability of better spectral hardware requires a much higher speed in acquisition, smart data elaboration, new set-ups, and new ideas, in order to make HSI tools compatible with real time needs, for instance looking for food contamination or for obtaining prompt cancer detection along routine medical checks.

Mathematical tools and algorithms are also needed for HSI data across many different applications, facilitating chemo-physical identification of materials on the basis of their unique spectral signature that is usually buried under a huge amount of data.

The aim of this Special Issue is to offer a view of current topics that are believed to become more significant in the future, hoping that the methods of acquisition and analysis shown in the articles can be used as template cases or for a better understanding of a large variety of problems.

## 2. Contributions

Hyperspectral imaging offers great opportunities, but it requires careful handling of information obtained in order to have a clear vision of the spectroscopic signatures that might become hidden within a huge amount of data. Moreover, different sources of signals can be obtained simultaneously to each other and a problem arises in how to meaningfully merge the different contributions.

Therefore, there are contributions related to fuse different spectral bands or deal with signals originating from diverse decay channels (e.g., luminescence and Raman).

Likewise, in order to reduce the effort, various compression strategies in the spectroscopic realm are well illustrated.

More theoretical smart tools are then introduced and reviewed that are sophisticated and at the same time versatile so as to better investigate the intricacies of HIS hypercube in searching for specific spectroscopic signatures.

Various specific applications are presented as well. Aside from their specific application areas, their practical realization requires a high skill level from the technical set-up to the data analysis, to make the presentation more widely interesting.

In particular, there are experimental and theoretical contributions for time series observations, compression, and specific signatures, which are a very firm step toward 4D representation and usage of HSI data.

In Radi et al. [1], data spectral fusion is shown as coming from a Visible and Near Infrared VIS/NIR spectroscopic system and from a VIS/NIR hyperspectral imaging systems used respectively in interactance mode and in reflection mode with the aim of acquiring the spectral information of whole tubers for predicting the primordial leaf count of potatoes. In particular, the HSI unit uses an optical fiber to illuminate a large portion of the sampled potato with the imaging spectrograph directly attached to a CCD camera, acquiring spectral information along a line of the potato that is held by a scanner system, therefore in push broom modality.

Results inferred from this study, with the aid of partial least squares (IPLS), initiate the possibility of developing a portable or stationary electronic system aimed at obtaining a rapid and accurate prediction of the sprouting activity of stored potatoes. Future steps could be in testing more growing seasons for many cultivated varieties (cultivars), as well as improving the robustness and reproducibility of the prediction models. Furthermore, an increase in the productivity of the method could be achieved through improved data elaboration, for instance reducing the number of selected wavelengths, and other IPLS models, such as moving average IPLS, synergy IPLS, backward/forward IPLS, or else a genetic algorithm.

In Bonifazi et al. [2], a HSI set-up is used to evaluate the stability against light and UV ageing of several painting materials, in particular powder pigments and commercial watercolors to be used in retouching. In order to obtain HSI data, a pushbroom commercial system acquires hyperspectral images in the short-wave infrared region, in the spectral wavelength interval between 1000 nm and 2500 nm. The pixel resolution by scanning the sample is between 30 micron and 300 micron, building the image one line at a time while the sample is scanned on a moving sample tray in front of the camera. The paper shows the possibility of evaluating minute spectral variations due to ageing times well before the eyes can detect changes: The classification techniques based on principal component analysis (PCA) and k-nearest neighbor (KNN) of hyperspectral data are effectively capable of monitoring the changes occurring in the painting layers. Therefore, HSI coupled with a chemometric approach allows one to monitor ageing modifications of paint layers, showing the possibility of detecting damages before its irreversibility, a result of great relevance in the field of cultural heritage and particularly useful in monitoring artworks and restoration interventions over time at a lower cost compared to similar methods. Future research lines are suggested, for instance in studying other restoration materials, like synthetic resins, or the classification and forecast of material behavior involved in cultural heritage artifacts.

In Bachmann et al. [3], hyperspectral image sequences are considered for the detection and tracking of vehicles realizing low-rate video hyperspectral imaging systems. The vehicles are assumed to be driving through the parking lot and passing behind various occlusions within the scene, such as trees in the background and parked cars, at a specified maximum speed. The set-up includes an integration of a high rate data acquisition hyperspectral line scanner with a high-speed maritime pan-tilt unit that contains position and pointing information. Then, the realization of digital elevation models (DEM) is devised: HSI time series imagery after integration onto a telescopic pole system from multiple vantage points, stereo hyperspectral views, and the definition of bi-directional reflectance distribution function can all be derived. Two examples of the low-rate hyperspectral video approach are shown, that is the imaging of the dynamics of the surf zone in a coastal setting and moving vehicle imaging in the presence of many occlusions. For future, the authors propose further improvements in hyperspectral image acquisition rates by reducing the size of the across-track spatial dimension or the adoption of already commercially available sensors with on-chip spectral binning.

Yang et al. [4], explore the medical implications of predicting the healing of diabetic foot ulcers by a HSI instrument. Such a tool is presented as highly helpful because foot ulcers are indeed a major complication of diabetes. Previously, the biomedical application of HSI has been able to anticipate wound healing based on SpO_2_ values due to the different absorption spectra of oxy- and deoxyhemoglobin. Here, PCA is addressed as an alternative approach to improving the prediction of wound healing. A comparison is also made with the performance of SpO_2_ mapping. It is found that the PCA second principal component elaborated on hyperspectral images appears superior to analysis in comparison to SpO_2_ values in predicting the healing of wounds, taken at a baseline of 12 weeks. A HSI camera operating in pushbroom mode, for which the images are taken one line at a time from the scene, is part of the set-up that include a CCD camera coupled to an imaging spectrograph. Each 3D data cube taken in this study, after a sweep from heel to toe, is reduced to simpler regions of interest of 50 pixels × 50 pixels, for which the authors found enough information to characterize the wound and surrounding tissue for all obtained images. The PC analysis is deeply discussed, pointing to PC2 discrimination in the oxy- and deoxyhemoglobin spectra with superior performance to SpO_2_ measurement strategies.

Behmann et al. [5], address the problem of characterization of plant disease. A method is presented to spatially reference the time series of a close range of hyperspectral images, overcoming the inability of previous studies of symptoms over time to detect early stages of a disease when they are still invisible. Automatically tracked hyperspectral information could be a promising approach to overcome this limitation.

Results have been shown for the first symptoms and their development in the presence of septoria tritici blotch (STB) and brown rust as well, by using a VISNIR camera. The tracking of symptoms in real space is obtained measuring reflectivity from plants between 400 nm and 1000 nm in pushbroom mode, and, then, making a space reference on multiple hyperspectral image cubes along the time series direction. Those spatially referenced images form therefore a new 4D data type with two spatial axis, one spectral axis, and a fourth temporal axis (x, y, λ, t). Within this new data type, disease symptoms can easily be traced back in time, even to the point when no symptom is visible to the human eye. The point correspondence problem is solved through referencing by including the RANSAC algorithms and multiple 2D geometric transformations in combination with a well-defined set of control points.

The possibility to annotate invisible symptoms by tracing visible symptoms back in time to the invisible phase of pathogenesis shows that automated referencing of hyperspectral images is possible. The training of machine learning models will also allow higher sensitivity even at the very early symptom stages. The claim is that this is a really new approach in hyperspectral series imaging, moving the focus from mature symptoms and their appearing in the visible bands to very early and invisible stages of plant diseases.

Shen et al. [6], have proposed a new predictive lossless compression algorithm for multiple time series of time-lapse hyperspectral image data using a low-complexity sign algorithm with an expanded prediction context. It is noted that HSI technology has been used for various remote sensing applications due to its excellent capability of monitoring regions-of-interest over a period of time. Simulation results have demonstrated the outstanding capability of this algorithm to compress a temporal series of HSI data through spectral and temporal decorrelation. The actual compression results are congruent with the information theoretic analysis and estimation based on conditional entropy. The paper is about an information theoretical analysis to estimate the potential compression performance gain with varying configurations of context vectors. In fact, it shows how compression performance varies as a function of the initial set of spectral bands for prediction by exploiting the spectral and temporal correlations in the datasets. As examples of future work, the authors propose a full integration of the proposed algorithm and the analytic framework to achieve real-time compression on streaming hyperspectral images, including an adaptive selection of bands to build up an optimal context vector data. By extending a lossless compression of regions-of-interest in hyperspectral images, it is also possible to gain a much higher compression than compressing the entire hyperspectral image dataset.

In Cadd et al. [7], a novel application of visible wavelength reflectance HSI is shown for both detection and age determination of blood-stained fingerprints on white ceramic tiles based on the signature of hemoglobin in the visible absorption spectrum between 400 and 680 nm and for the presence of a Soret peak at 415 nm. Blood-stained fingerprints were aged for over 30 days and analyzed using HSI. Data produced results organized on a 24 h scale and a 30-day scale. A clear age estimation of deposited blood-stained fingerprints has been shown to be therefore possible, while a similar visual examination was not possible using a standard digital photographic camera. The HSI system used in this study had the same setup as a previous one shown by the same authors consisting in a liquid crystal tunable filter (LCTF), coupled to a digital camera, and a scene illumination with two LED light sources, for VIS and UV. Control of the LCTF and the entire process by means of a custom software takes approximately 30 s to acquire and process each image, demonstrating that HSI could be used for the detection and identification of both blood stains and blood-stained fingerprints together with determining their age. More rugged and portable instruments for use at crime scenes could be realized in the future, which would be particularly beneficial for criminal investigations.

In Arablouei [8], a new algorithm is proposed capable of simultaneously fusing multiple multiband HSI images. The used method relies on a forward observation model together with a linear mixture model. The low rank images produced will have a sparse representation in the spectral domain, while preserving the edges and discontinuities in the spatial domain in agreement with the fact that HSI image data are generally known to have a low-rank structure. It is noted, in fact, that, due to correlations among the spectral bands and the fact that the spectrum of each pixel can often be represented as a linear combination of relatively few spectral signatures, the images reside in a subspace that usually has a much smaller dimension than the number of the spectral bands. As a result, it is possible to decompose linearly a hyperspectral image into its constituent endmembers, spectral signatures of the material present at the scene, down to fractional abundances of the endmembers for each pixel. This linear decomposition is what the authors define as spectral unmixing and the corresponding data model the linear mixture model. A comparison with the state-of-the-art fusion methods is made in this paper, demonstrating the advantages of the proposed algorithm. In particular, they show results from experiments with five real hyperspectral images that were done following the Wald’s protocol, a general paradigm for quality assessment of fused images regarding their consistency and synthesis properties.

In Gruber et al. [9], a fast line-scanning hyperspectral imaging system is described. The experiments carried out, shown by four different applications, demonstrate that a Laser-excited HSI system makes possible the acquisition of Raman and fluorescence spectra on relatively large sample areas, with fast and high spatial resolution scans. As it is clear and well known, it is not easy to detect the weaker Raman signals compared to the higher fluorescence background as the two are competitive and simultaneous. However, the observation is made that HSI only requires the highest possible spectral variance data to work well for evaluation or classification purposes, so the exact knowledge of the origin or a localized distinction of the signals is of secondary importance. The set-up presented opens up interesting application possibilities in many areas. In fact, the modular design of the system makes it possible to adapt the measuring range and spatial resolution to many different application areas, from quality control in the food industry to surface inspection and recycling. The Laser-HSI system, as it is called in the paper, operates as a pushbroom imager with the hypercube generated line by line while the sample is scanned using a linear motion unit over tens of centimeters to millimeter ranges, with a ad-hoc alignment of the dichroic mirror to achieve a balanced Raman and fluorescence intensities. Classification of the measurements using machine learning algorithms was also demonstrated, after a careful spectral calibration against reference substances. Results support the idea that Laser-HSI can be used for various applications in the field of process and food monitoring or sorting tasks just where conventional HSI systems fail.

The merge of HSI and deep learning (DL) technologies is the subject of a review made by Signoroni et al. [10]. HSI data constitutes an undeniable advantage for any research that benefits from computer-assisted spectroscopic analysis. In fact, HSI images have plenty of information coded that can be thought as a high-dimensional vector in a space and spectral dimension with much more information than any RGB or multispectral data. Each pixel can keep measurements in relatively wide spectral intervals, from VIS to NIR, resolved in hundreds of contiguous narrow band spectral channels down to a few nm of spectral resolution. However, as any industrial or scientific technology, HSI requires cost-benefit evaluations and any method to unlock its deployment potentialities is important and needs careful consideration. As explained by the authors, the advantages introduced by DL solutions in the HSI arena are in the automatic and hierarchical learning process of data itself. A model with increasingly higher semantic layers can then be built, until the searched analysis, e.g., classification, regression, segmentation, detection, or other indexes, has a useful representation. Some caution is however needed to exploit the gain potentially detained by DL when it is applied to hyperspectral data, as pointed out in this review. In particular, there is a need for a reasonably large dataset in HSI data (e.g., hundreds of thousands of examples) that has to be congruent with the large amount of parameters of DL models (typically in the tens of millions), to avoid overfitting, while an HSI dataset composed of hundreds of examples can be considered too small. In conclusion, the deployment of HSI technologies by means of DL solutions can be a possible driver enabling HSI for a wider spectrum of small-scale applications in industry, biology and medicine, cultural heritage, and other professional fields.

A review by Oiknine et al. [11], shows the advances of a specific HSI system, the compressive sensing miniature ultra-spectral imaging (CS-MUSI) camera. This article provides an evaluation of the CS-MUSI camera, its evolution, and its different applications. The CS-MUSI camera has been designed for using a liquid crystal (LC) phase retarder in order to modulate the spectral domain, realizing therefore a spectral compression. The outstanding advantage of the CS-MUSI camera is that at least one order of magnitude of fewer measurements are needed for the entire HSI image results in comparison with conventional HSI images, as a consequence that the scene’s spectral properties are often redundant in nature. This paper shows the reconstruction of HSI images for both cases when the camera and scene are stationary as well as for when the camera is moving in the along-track direction, demonstrating the ability to use the CS-MUSI camera for 4D spectral-volumetric imaging. Experiments in these scenarios and applications have provided a spectral uncertainty of less than one nanometer. Alternatively, this method can also be realized with other spectral modulators. Other compressive methods, like Fabry-Perot resonator (mFPR), which has a much faster response time than LC cells, and snapshot HS camera, by using parallel spectral multiplexing and including an array of mFPRs and a lens array, are presented in the extensive references of the same authors, thus completing the topic.

Finally, compressing Raman HSI data is reviewed in Cebeci et al. [12]. As noted by the authors, a fast Raman analysis for real-time monitoring and hyperspectral imaging has a key bottleneck in the time required to acquire and post-process HSI data. Multichannel detectors (CCD) are commonly used for this task, although they are generally more expensive and less sensitive than single channel detectors. The CCDs also require cooling because of the usual need for long integration times and low dark counts. Consequently, a CCD-based Raman spectrometer cannot operate fast enough to be applicable to dynamic system measurements. Using ordinary Raman HSI spectroscopy, where thousands to millions of different spatial points are measured, would imply the collection of a one-megapixel image over 12 days using 1 s of acquisition rate per spectrum, clearly an impossible task. Instead, a compressive spectrometer with spatial light modulator (SLM) technology offers higher sensitivity and speed, together with a lower-cost alternative to CCDs because of the single detector adoption. Most notably, the compressive detection allows chemical imaging information in the very low signal limit, which is simply impossible to achieve by a conventional CCD-based Raman spectroscope. The chosen key technology is an optimized binary compressive detection strategy (OBCD) adopting a reflective light modulator DMD (by Texas Instruments), widely used in standard computer projection systems. A DMD is a semiconductor-based “light switch” array of hundreds of thousands of individually switchable mirror pixels, which have switch speed, contrast ratio, and broad spectral capability outperforming analog-based SLMs. The light switching speeds in the order of kHz at which each mirror can modulate between “on” and “off” states enable CD measurements at kHz frequencies. Compressive digital (CD) Raman systems can reproduce the functionality of conventional array-based Raman spectroscopy to collect full spectral information by raster-scanning each array column. The full speed advantage of CD Raman is fully discussed in relation to the compressive detection modes, with filter functions, and a low signal regime or high-speed conditions, all conditions for which a CCD cannot work, while a single channel detector, such as a photomultiplier, instead increases dramatically data SNR.

## 3. Conclusions

Hopefully it is clear, from the lecture of the various contributions, that all authors have realized a very serious effort in devising the perspective of their work in a broader realm than the proper single activity.

Various compression and fusion methods, coupled with time series HSI acquisitions, embedded in several real-world examples and theoretical tools, make this Special Issue “The Future of Hyperspectral Imaging” a very interesting instrument to better understand the possible future of this versatile technique.

## References

[1] Rady A., Guyer D., Kirk W., Donis-González I.R. (2019). Prediction of the Leaf Primordia of Potato Tubers Using Sensor Fusion and Wavelength Selection. J. Imaging.

[2] Bonifazi G., Capobianco G., Pelosi C., Serranti S. (2019). Hyperspectral Imaging as Powerful Technique for Investigating the Stability of Painting Samples. J. Imaging.

[3] Bachmann C.M., Eon R.S., Lapszynski C.S., Badura G.P., Vodacek A., Hoffman M.J., McKeown D., Kremens R.L., Richardson M., Bauch T. (2019). A Low-Rate Video Approach to Hyperspectral Imaging of Dynamic Scenes. J. Imaging.

[4] Yang Q., Sun S., Jeffcoate W.J., Clark D.J., Musgove A., Game F.L., Morgan S.P. (2018). Investigation of the Performance of Hyperspectral Imaging by Principal Component Analysis in the Prediction of Healing of Diabetic Foot Ulcers. J. Imaging.

[5] Behmann J., Bohnenkamp D., Paulus S., Mahlein A.-K. (2018). Spatial Referencing of Hyperspectral Images for Tracing of Plant Disease Symptoms. J. Imaging.

[6] Shen H., Jiang Z., Pan W.D. (2018). Efficient Lossless Compression of Multitemporal Hyperspectral Image Data. J. Imaging.

[7] Cadd S., Li B., Beveridge P., O’Hare W.T., Islam M. (2018). Age Determination of Blood-Stained Fingerprints Using Visible Wavelength Reflectance Hyperspectral Imaging. J. Imaging.

[8] Arablouei R. (2018). Fusing Multiple Multiband Images. J. Imaging.

[9] Gruber F., Wollmann P., Grählert W., Kaskel S. (2018). Hyperspectral Imaging Using Laser Excitation for Fast Raman and Fluorescence Hyperspectral Imaging for Sorting and Quality Control Applications. J. Imaging.

[10] Signoroni A., Savardi M., Baronio A., Benini S. (2019). Deep Learning Meets Hyperspectral Image Analysis: A Multidisciplinary Review. J. Imaging.

[11] Oiknine Y., August I., Farber V., Gedalin D., Stern A. (2019). Compressive Sensing Hyperspectral Imaging by Spectral Multiplexing with Liquid Crystal. J. Imaging.

[12] Cebeci D., Mankani B.R., Ben-Amotz D. (2019). Recent Trends in Compressive Raman Spectroscopy Using DMD-Based Binary Detection. J. Imaging.

